# Venous thromboembolism risk factors in pediatric patients with high-grade glioma: a multicenter retrospective study

**DOI:** 10.3389/fped.2025.1595223

**Published:** 2025-08-12

**Authors:** Yanxia Chen, Wenjing Fei, Yaqin Shi, Weiwei Ma, Wei Jiao, Fengqin Tao, Jie Zhu, Yuhai Wang, Xiaoyan Feng

**Affiliations:** ^1^ Department of Neurosurgery, The 904th Hospital of PLA, Medical School of Anhui Medical University, Wuxi, China; ^2^ Department of Critical Care Medicine, Institute of General Surgery, PLA Eastern Theater Command General Hospital, Nanjing, China; ^3^ Department of General Surgery, The 904th Hospital of PLA, Medical School of Anhui Medical University, Wuxi, China; ^4^ Department of Nursing, The 904th Hospital of PLA, Medical School of Anhui Medical University, Wuxi, China; ^5^ Department of Orthopedics, The 904th Hospital of PLA, Medical School of Anhui Medical University, Wuxi, China

**Keywords:** pediatric high-grade glioma, venous thromboembolism, risk factors, anticoagulation therapy, survival outcomes

## Abstract

**Background:**

Venous Thromboembolism (VTE) is a significant complication in pediatric high-grade glioma (pHGG) patients, impacting prognosis and treatment outcomes. Identifying unique risk factors and pathophysiology in children is essential for targeted prevention and treatment.

**Methods:**

A multicenter retrospective analysis was conducted on pHGG patients enrolled between January 2012 and January 2024 at two hospitals. Data were collected from electronic medical records and follow-ups, focusing on VTE occurrence, clinical characteristics, and treatment outcomes. Statistical analyses included *t*-tests, Mann–Whitney *U*-tests, chi-square tests, and Cox regression models to identify risk factors and their impact on survival.

**Results:**

Out of 216 screened patients, 168 met the inclusion criteria. The mean age was 9.87 ± 3.67 years, with 37.5% experiencing VTE. Tumor volume, grade, and specific genetic mutations significantly influenced VTE occurrence. Anticoagulation therapy, Isocitrate Dehydrogenase 1 (IDH1) mutations, O6-Methylguanine-DNA Methyltransferase (MGMT) methylation, radiotherapy, chemotherapy, and prolonged bed rest were protective against VTE, while increased tumor volume, Grade 4 glioma, Epidermal Growth Factor Receptor (EGFR) positivity, p53 mutations, glucocorticoid therapy and central venous catheter placement (CVCP) placement promoted VTE risk. The median survival time was 51.4 months, and VTE occurrence negatively impacted patient prognosis.

**Conclusion:**

This study highlights the risk factors for VTE in pHGG patients, emphasizing the need for tailored prevention and treatment strategies. The findings underscore the importance of clinical characteristics, genetic profiles, and treatment modalities in managing VTE and improving survival outcomes in pHGG.

## Introduction

Venous thromboembolism (VTE) is one of the most common paraneoplastic complications in cancer patients and a leading cause of cancer-related mortality (1, 2). It poses a significant burden on healthcare systems in terms of both cost and resource utilization, with glioma patients being particularly affected (3, 4). Studies have shown that up to 30% of glioma patients experience at least one VTE event during the course of their disease (5).

High-grade gliomas (HGG) accounts for 60%–70% of glioma occurrences. HGGs are highly vascular tumors, and concerns for increased risk of intracranial hemorrhage (ICH) due to therapeutic anticoagulation further complicate VTE management (6, 7). Additionally, patients with HGG typically undergo chemotherapy, and some agents, such as bevacizumab, may further influence coagulation status (8). Current evidence indicates that anticoagulation may increase the risk of ICH in patients receiving bevacizumab, although most events are asymptomatic, and uncontrolled case series suggest that the risk-benefit ratio favors anticoagulation therapy (9, 10).

The incidence of VTE in pediatric oncology patients is approximately 2.1%–16%, depending on the type of cancer. In the case of brain tumors, the VTE occurrence rate in children is quite low, ranging from less than 1 to 2.8%, which is lower than that in adult patients. However, these studies did not specifically focus on HGG. Compared to adult HGG, pediatric glioma patients exhibit significant differences in clinical symptoms, biological characteristics, pathogenesis, and treatment responses. Furthermore, children with HGG often have a poor prognosis, experiencing neurological dysfunction and are bedridden at an earlier stage. Additionally, these patients also require the use of chemotherapeutic drugs that can affect coagulation function. However, due to the vulnerability of the observed groups and other reasons, research on VTE in pediatric glioma patients is relatively scarce.

While several therapeutic options are available once VTE is diagnosed, and certain risk factors can guide prophylactic strategies, anticoagulants are neither without risk nor cost (11, 12). Moreover, damage from thromboembolic events is often irreversible, and ischemia-reperfusion injury can cause additional harm, underscoring the importance of VTE prevention (13). The risk factors for VTE in adult patients with HGG includes (14): (1) history of VTE; (2) hypertension; (3) asthma; (4) leukocyte count; (5) World Health Organization (WHO) tumor grade; (6) patient age; and (7) body mass index (BMI). Conversely, Isocitrate Dehydrogenase 1 (IDH1) mutation, hypothyroidism, and O6-Methylguanine-DNA Methyltransferase (MGMT) promoter methylation were identified as protective factors.

However, there is currently a lack of research on the risk factors for VTE specifically in pediatric patients with HGG. Given the scarcity of such studies and the necessity for further investigation, this study aims to systematically analyze the risk factors for VTE in pediatric HGG (pHGG) patients through a multi-center retrospective study, which will aim to provide more precise clinical strategies for the prevention and treatment of VTE in pHGG patients.

## Methods

### Objectives

Primary Objective: to identify VTE risk factors in pHGG patients through multicenter retrospective analysis. Secondary Objectives: 1. To assess the impact of VTE on survival outcomes in pHGG patients; 2. To explore the influence of various risk factors on survival outcomes.

### Patient cohort

This study was a multicenter retrospective analysis. Pediatric patients with HGG, treated at the 904th Hospital of PLA (Wuxi, Jiangsu, China) and the PLA Eastern Theater Command General Hospital (Nanjing, Jiangsu, China) between January 2012 and January 2024, were included. As this was a non-interventional study with no harm to patients, ethical exemption were granted by the Ethics Committee of the 904th Hospital of PLA (lead institution). Informed consent had been obtained during treatment, and all personal and sensitive data were anonymized. The study was conducted in accordance with the Declaration of Helsinki.

### Eligibility criteria

#### Inclusion criteria

(1). Patients under 18 years of age at the time of diagnosis; (2). Pathologically or molecularly confirmed HGG (Grade 3 or 4), including but not limited to anaplastic astrocytoma (AA), glioblastoma (GBM), diffuse midline glioma (DMG), and diffuse intrinsic pontine glioma (DIPG), as documented in institutional pathology reports; (3). Received standard or alternative tumor treatments (e.g., surgery, radiotherapy, chemotherapy, traditional Chinese medicine, targeted therapy, immunotherapy); (4). Documented VTE events or comprehensive thrombotic risk assessment data, or available VTE-related imaging or external records.

#### Exclusion criteria

(1). Unclear diagnosis without pathological or molecular confirmation; (2). Coexisting malignancies (e.g., leukemia, lymphoma); (3). Known congenital coagulation abnormalities (e.g., antithrombin deficiency, protein C or S deficiency); (4). Incomplete key clinical data (e.g., missing VTE records, pathology reports).

### Data collection

Data were primarily extracted from electronic medical records of both hospitals. Follow-up data were obtained via telephone or video calls. All data were entered into anonymized Excel spreadsheets.

Primary data collected: VTE occurrence. Secondary data collected: Age, gender, survival status (survival time/follow-up duration), anticoagulation therapy administration, incidence of bleeding complications, tumor volume and grade, genetic mutations, treatment, immobilization status, glucocorticoids use, and history of central venous catheter placement (CVCP).

Definitions of Study Variables: VTE: Diagnosed via Doppler ultrasound indicating venous flow obstruction or thrombus; or computed tomography venography (CTV) showing intraluminal filling defects; or magnetic resonance venography (MRV) confirming venous thrombosis. Tumor grading: Based on institutional pathology reports. Prolonged immobilization: Continuous bed rest exceeding two weeks. Tumor volume: Calculated as diameter × diameter at the largest cross-sectional area.

### Statistical analysis

Primary Endpoint: Risk factors associated with VTE occurrence. Secondary Endpoints: Relationship between VTE and survival outcomes; influence of various clinical variables on survival.

Data analysis was performed using SPSS 22.0. Continuous variables were expressed as mean ± standard deviation or median (interquartile range), and categorical variables as frequencies (percentages). Depending on normality, continuous variables were compared using *t*-tests or Mann–Whitney *U*-tests, while categorical variables were assessed using chi-square tests. VTE risk factors were evaluated via univariate or multivariate Cox regression analysis. Correlation of continuous variables was analyzed by linear regression. Statistical significance was set at *p* < 0.05.

## Results

### Clinical characteristics at admission

**Figure 1:**
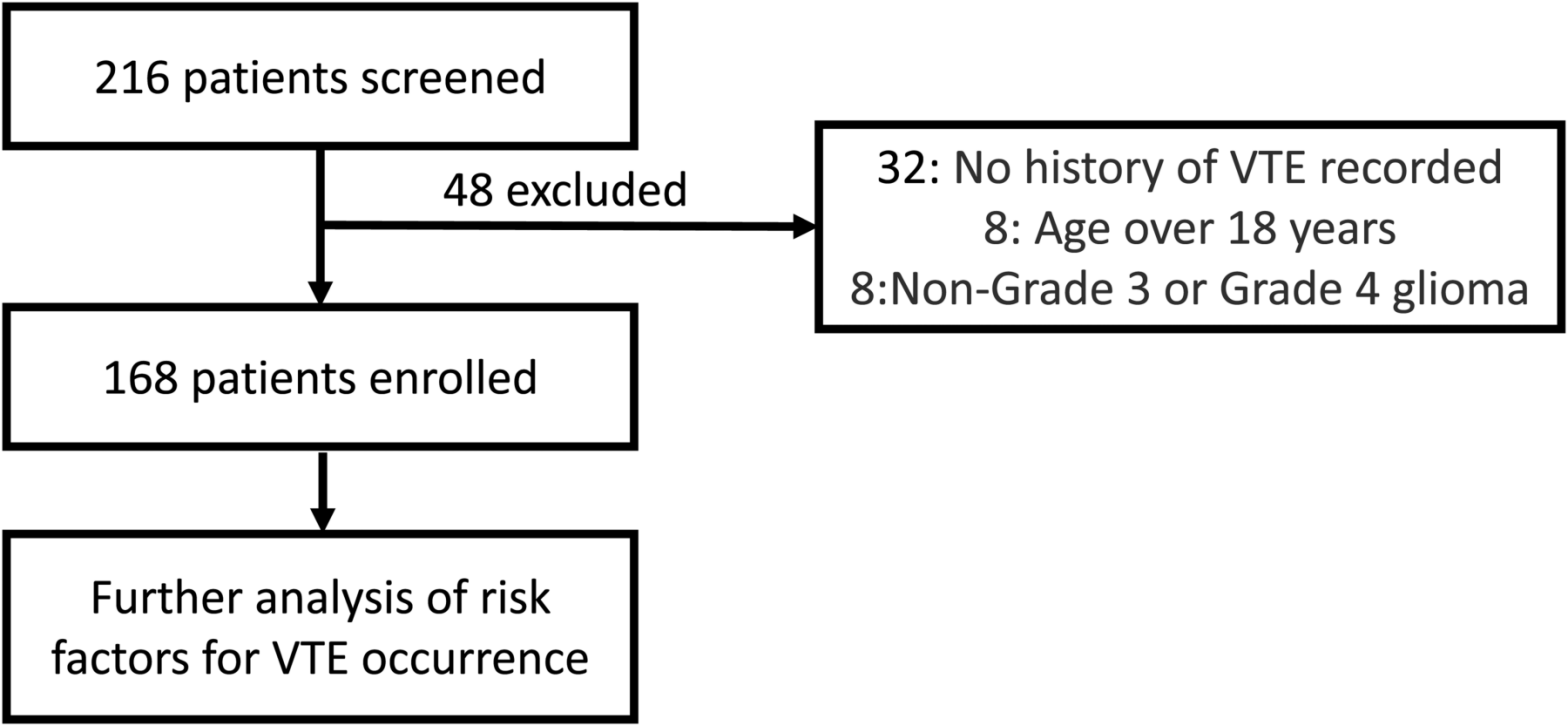
This flowchart illustrates the patient screening process for this study. 216 patients were initially screened, of which 48 were excluded, resulting in 168 enrolled patients. 32 patients were excluded due to no history of VTE assessed, 8 patients were excluded for being over 18 years of age, and 8 patients were excluded for having non-Grade 3 or non-Grade 4 glioma.

A total of 216 patients were initially screened for this study. Of these, 48 were excluded for not meeting the inclusion criteria: 32 patients lacked definitive documentation of VTE, 8 patients were over 18 years old at admission, and another 8 had diagnoses other than HGG (Figure 1). Ultimately, 168 patients were included in the final analysis. Table 1 summarizes the clinical characteristics of the enrolled cohort. The mean age was 9.87 ± 3.67 years, with a lower proportion of females (48.21%). VTE occurred in 37.5% of patients. Among 108 patients with available data, 14.81% received anticoagulant therapy.

**Table 1:** Clinical characteristics of patients at admission.

Clinical characteristics	*n*	*n* (%/Mean ± SD^a^)
Age (y)	168	9.87 ± 3.67
Gender (female)	168	81 (48.21%)
VTE occurrences	168	63 (37.5%)
Anticoagulation therapy	108	16 (14.81%)
Tumor volume (cm^2^)	119	28.47 ± 9.27
Tumor grade^b^	168	
3		91 (54.17%)
4		77 (45.83%)
Gene expression	121	
IDH1 MUT		20 (16.53%)
ATRX^+^		8 (6.61%)
EGFR^+^		29 (23.97%)
MGMT methylation		39 (32.23%)
p53 MUT		23 (19.01%)
Treatment	147	
Radiotherapy		119 (80.95%)
Chemotherapy		59 (40.69%)
Target therapies		12 (8.16%)
Chinese traditional medicine		11 (7.48%)
Prolonged bed rest^c^	113	105 (92.92%)
Glucocorticoid therapy	152	64 (42.11%)
History of CVCP	146	12(8.22%)

VTE, venous thromboembolism; IDH1 MUT, isocitrate dehydrogenase 1 mutation; ATRX +, alpha-thalassemia/mental retardation syndrome X-linked positive; EGFR +, epidermal growth factor receptor positive; MGMT, O6-methylguanine-DNA methyltransferase; p53, tumor protein p53; CVCP, central venous catheter placement.

^a^ Categorical variables are presented as percentages, while continuous variables are presented as mean ± standard deviation.

^b^ According to the retained pathological reports.

^c^ Bed rest for more than 2 weeks.

The average tumor volume among 119 patients was 28.47 ± 9.27 cm^2^. Tumor grading revealed that 54.17% had Grade 3 gliomas, and 45.83% had Grade 4. Genetic profiling when available showed IDH1 mutations in 16.53% of patients, Alpha-Thalassemia/Mental Retardation (ATRX) positivity in 6.61%, Epidermal Growth Factor Receptor Positive (EGFR) positivity in 23.97%, MGMT promoter methylation in 32.23%, and p53 mutations in 19.01%.

Regarding treatment, 80.95% of patients received radiotherapy, 40.69% underwent chemotherapy, 8.16% received targeted therapies and 7.48% received traditional Chinese medicine. Among 113 patients with data on mobility, 92.92% experienced prolonged bed rest. Of 152 patients, 42.11% were treated with corticosteroids, and 8.22% of 146 patients had a history of CVCP.

### Comparison of clinical characteristics based on VTE status

The classification of VTE events has been documented in Table 2 demonstrating a clear predominance of DVT, accounting for 90.5% (57/63) of all VTE cases, while pulmonary embolism (PE) represented only 9.5% (6/63) of thrombotic events.

**Table 2:** Classification of VTE events in pediatric HGG.

VTE type and location	Cases (*n* = 63)
DVT only	57 (90.5%)
Proximal lower extremity (femoral/iliac vein)	38 (66.7%)
Distal lower extremity (popliteal/tibial vein)	16 (28.1%)
Upper extremity veins	3 (5.3%)
PE	6 (9.5%)
PE with concurrent DVT	5 (83.3%)
Isolated PE (no DVT)	1 (16.7%)

VTE, venous thromboembolism; HGG, high grade giloma; DVT, deep vein thrombosis; PE, pulmonary embolism.

Patients were stratified according to VTE status for comparative analysis (Table 3). There was no significant difference in mean age between the VTE group (10.01 ± 3.27 years) and the non-VTE group (9.84 ± 3.91 years; *p* = 0.62). However, gender distribution differed significantly, with females comprising 33.33% of the VTE group vs. 57.14% of the non-VTE group (*p* < 0.001). There was no significant difference in anticoagulant use between the two groups (5.88% vs. 18.92%; *p* = 0.14). The tumor volume in the VTE group was significantly larger than in the non-VTE group (34.54 ± 5.58 cm^2^ vs. 24.97 ± 9.02 cm^2^; *p* < 0.001). Tumor grade also differed notably, with 90.48% of the VTE group having Grade 4 tumors compared to 19.05% in the non-VTE group (*p* < 0.001).

**Table 3:** Comparison of clinical information based on VTE occurrence.

Clinical information	VTE^a^	No VTE^a^	*p*
Age (y)	10.01 ± 3.27	9.84 ± 3.91	0.62
Gender (female)	21 (33.33%)	60 (57.14%)	<0.001
Anticoagulation therapy	2/34 (5.88%)	14 (18.92%)	0.14
Tumor volume (cm^2^)	34.54 ± 5.58	24.97 ± 9.02	<0.001
Tumor grade^b^			<0.001
3	6 (9.52%)	85 (80.95%)	
4	57 (90.48%)	20 (19.05%)	
Gene Expression			
IDH1 MUT	3 (6.52%)	17 (22.67%)	<0.001
ATRX +	5 (10.87%)	3 (4.00%)	0.27
EGFR +	14 (30.43%)	15 (20.00%)	0.28
MGMT Methylation	14 (30.43%)	25 (33.33%)	0.90
p53 MUT	12 (26.09%)	11 (14.67%)	0.19
Treatment			
Radiotherapy	39 (65.00%)	80 (91.95%)	<0.001
Chemotherapy	18 (30.00%)	41 (48.24%)	<0.001
Target Therapies	4 (6.67%)	8 (9.20%)	0.78
Chinese Traditional Medicine	5 (8.33%)	6 (6.90%)	0.77
Prolonged Bed Rest^c^	45 (86.54%)	60 (98.36%)	<0.001
Glucocorticoid Therapy	34 (56.67%)	30 (32.61%)	<0.001
History of CVCP	2 (3.33%)	10 (11.63%)	0.14

VTE, venous thromboembolism; IDH MUT, isocitrate dehydrogenase mutation; ATRX +, alpha-thalassemia/mental retardation syndrome X-linked positive; EGFR +, epidermal growth factor receptor positive; MGMT, O6-methylguanine-DNA methyltransferase; p53, tumor protein p53; CVCP, central venous catheter placement.

^a^ Categorical variables are presented as percentages, while continuous variables are presented as mean ± standard deviation.

^b^ According to the retained pathological reports.

^c^ Bed rest for more than 2 weeks.

Genetic analysis revealed a significant difference in IDH1 mutation rates (6.52% in the VTE group vs. 22.67% in the non-VTE group; *p* < 0.001). No significant differences were observed in ATRX +, EGFR +, MGMT Methylation, or p53 mutation between the groups. Treatment modalities varied significantly: the VTE group had lower rates of radiotherapy (65% vs. 91.95%; *p* < 0.001) and chemotherapy (30% vs. 48.24%; *p* < 0.001) compared to the non-VTE group. Target therapies and Chinese traditional medicine showed no significant difference between groups. Prolonged bed rest was more common in the non-VTE group (98.36% vs. 86.54%; *p* < 0.001), whereas corticosteroid use was higher in the VTE group (56.67% vs. 32.61%; *p* < 0.001). There was no significant difference in CVCP placement history between the groups.

To further explore VTE risk factors, univariate and multivariate Cox regression analyses were conducted (Table 4). Anticoagulant therapy, IDH1 mutation, MGMT methylation, radiotherapy, chemotherapy and prolonged bed rest were associated with reduced VTE risk. Conversely, larger tumor volume, Grade 4 glioma, EGFR positivity, p53 mutation, CVCP placement and corticosteroid use were identified as risk factors for VTE development. In addition, different chemotherapy regimens have no significant impact on the occurrence of VTE (Supplementary Table S1).

**Table 4:** Comparison of clinical information based on VTE occurrence.

Clinical information	OR (Univariate Analysis)	OR (Multivariate Analysis)
Age (y)	1.35	1.92
Gender (female)	0.98	4.05
Anticoagulation therapy	0.01	0.02
Tumor volume (cm^2^)	1.13	2.27
Tumor grade^a^	518.79	697.12
Gene expression		
IDH1 MUT	0.32	0.06
EGFR^+^	1.93	7.38
MGMT Methylation	0.49	0.12
p53 MUT	4.34	7.97
Treatment		
Radiotherapy	0.61	0.59
Chemotherapy	0.21	0.12
Target therapies	0.94	0.80
Chinese traditional medicinE	0.88	0.89
Prolonged bed rest^b^	0.36	0.01
Glucocorticoid therapy	9.41	6.24
History of CVCP	40.27	0.86

VTE, venous thromboembolism; IDH MUT, isocitrate dehydrogenase mutation; ATRX +, alpha-thalassemia/mental retardation syndrome X-linked positive; EGFR +, epidermal growth factor receptor positive; MGMT, O6-methylguanine-DNA methyltransferase; p53, tumor protein p53; CVCP, central venous catheter placement.

^a^ According to the retained pathological reports.

^b^ Bed rest for more than 2 weeks.

**Figure 2:**
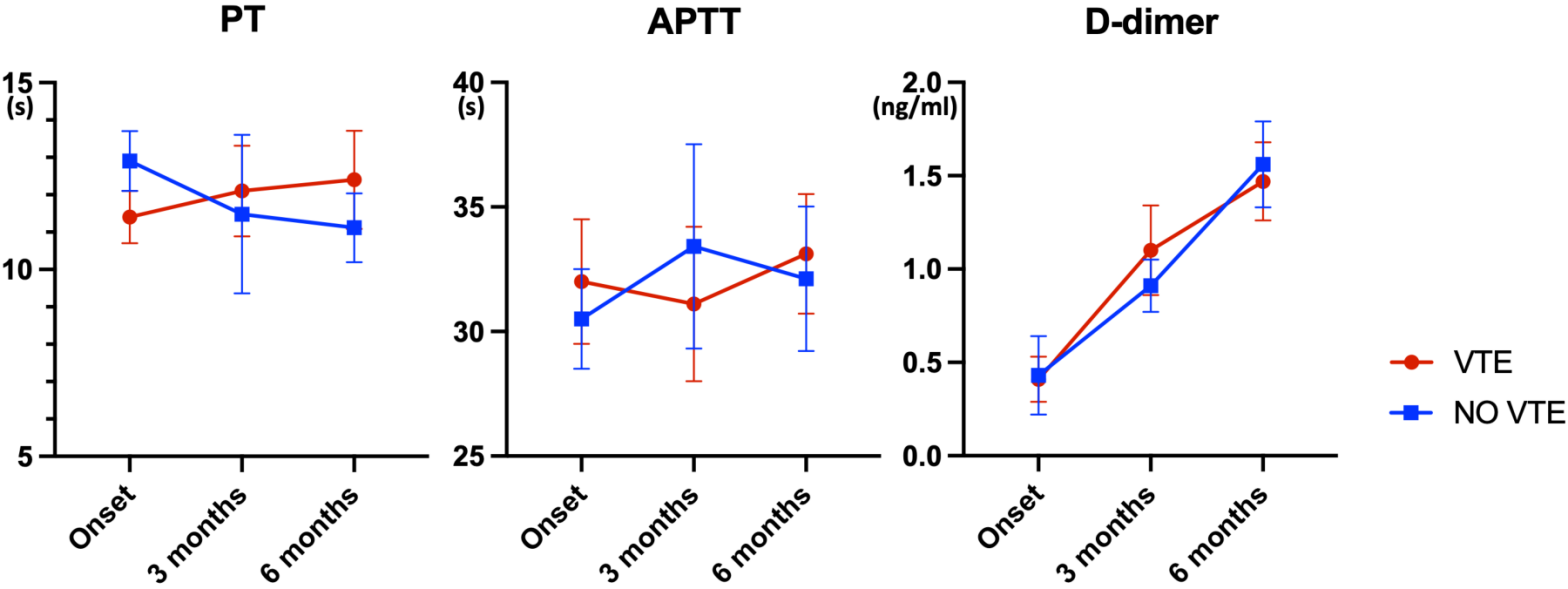
Temporal trends in coagulation parameters among glioma patients with and without VTE (*n* = 78, * indicates *p* < 0.05). This figure presents the Prothrombin Time (PT), Activated Partial Thromboplastin Time (APTT), and D-dimer levels measured at disease onset (diagnosis) and at 3 and 6 months post-onset in patients with and without venous thromboembolism (VTE). The data indicate no significant differences in PT and APTT between the two groups. However, D-dimer levels increased over time in both groups, with no significant intergroup differences observed.

We analyzed the Prothrombin Time (PT), Activated Partial Thromboplastin Time (APTT), and D-dimer levels of patients at the time of disease onset (diagnosis), as well as at 3 months and 6 months post-onset (Figure 2). There were no significant differences in PT and APTT between the two groups. However, the D-dimer levels increased over time in both groups, and there were still no significant differences between the groups.

### Survival analysis based on clinical characteristics

**Figure 3:**
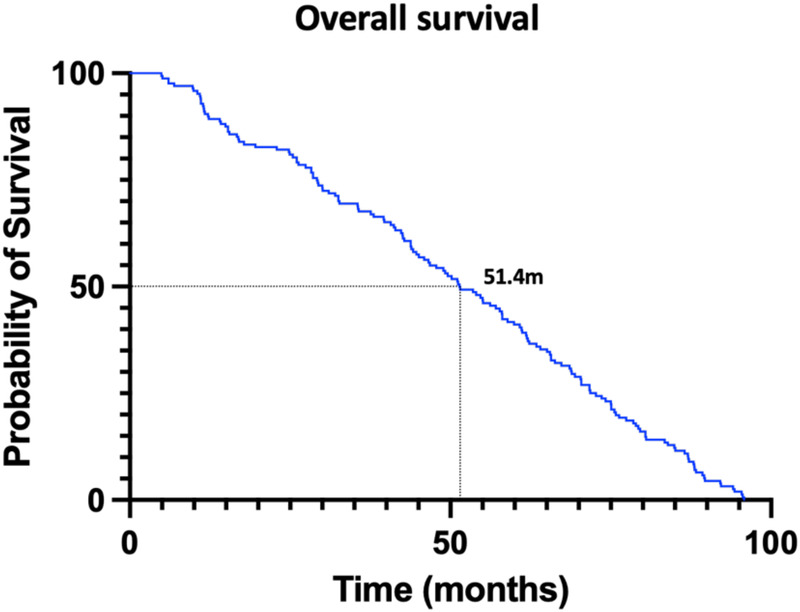
This figure represents the overall survival condition of pediatric patients of high grade glioma. (The Kaplan–Meier curve was used for analysis.) The median survival time was 51.4 months.

**Figure 4:**
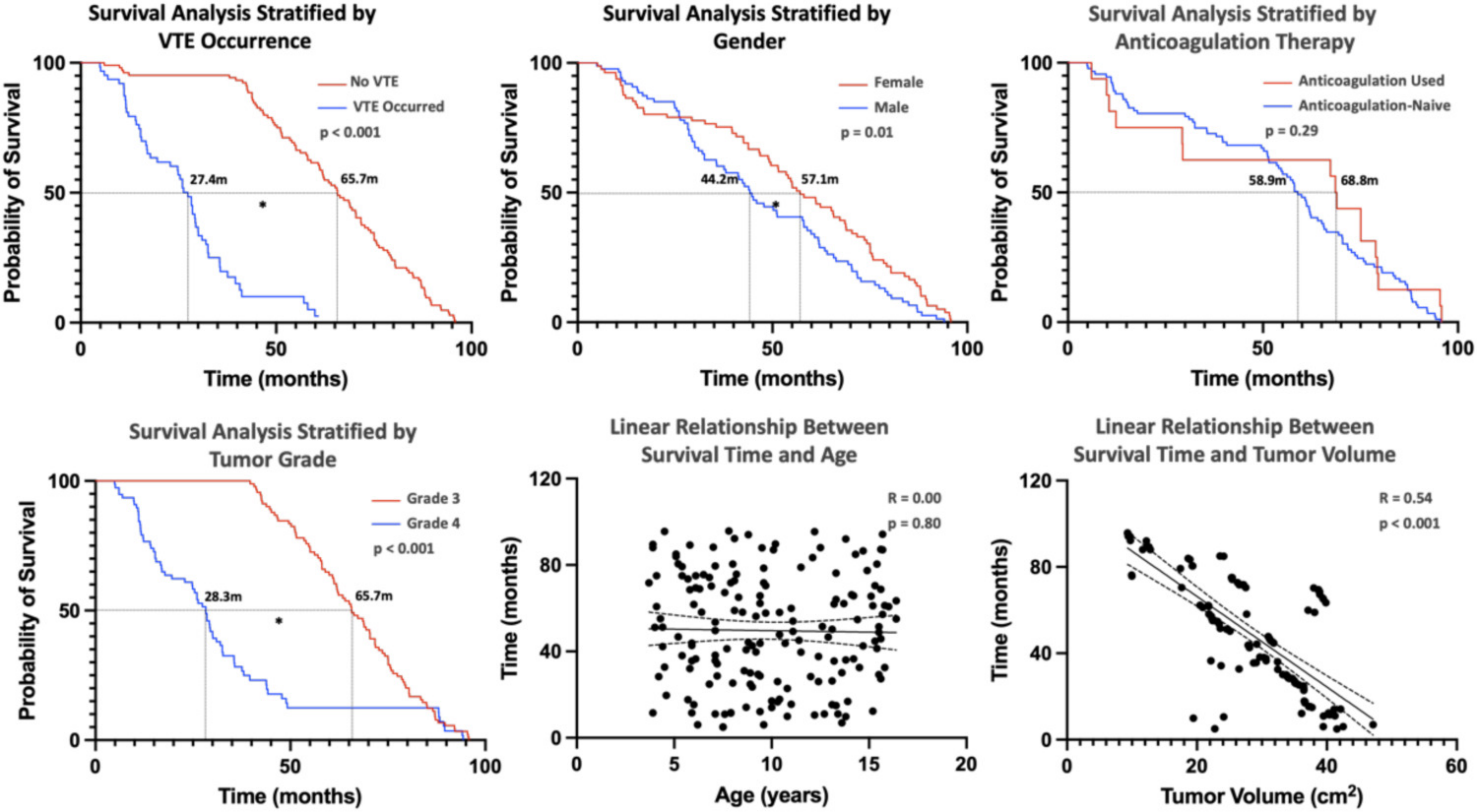
This figure represents the overall survival condition of pediatric patients with high-grade glioma stratified by clinical characteristics. [Kaplan–Meier (KM) curves were used for the analysis of categorical variables, and linear regression was used for continuous variables, with * indicating significance at *p* < 0.05.] The absence of VTE, female gender, and tumor grade (Grade 3) are favorable factors for patient survival and are statistically significant, while the use of anticoagulant therapy is not associated with patient survival time. Linear regression shows no association between patient survival time and age, but a negative correlation with tumor volume.

Survival analysis of pHGG patients identified several key prognostic factors. Figure 3 presents overall survival, with a median survival of 51.4 months determined via Kaplan–Meier (KM) analysis. Stratified analysis was performed based on clinical characteristics (Figure 4), utilizing KM curves for categorical variables and linear regression for continuous variables. Absence of VTE, female sex, and lower tumor grade (Grade 3) were significantly associated with improved survival, while anticoagulant therapy did not significantly influence survival outcomes.

**Figure 5:**
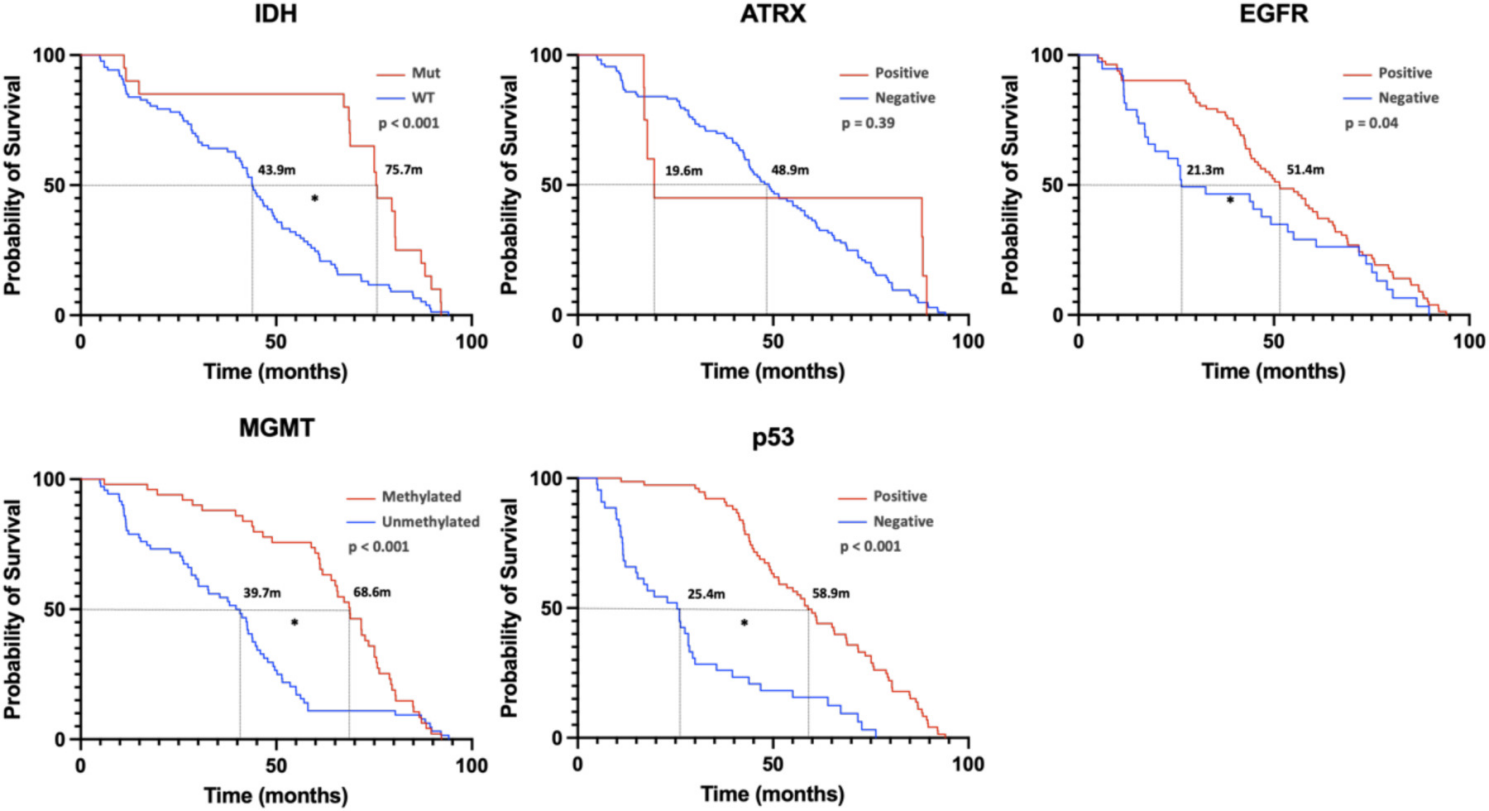
This figure represents the overall survival condition of pediatric patients with high-grade glioma stratified by gene expression. [Kaplan–Meier (KM) curves were used for the analysis, with * indicating significance at p < 0.05.] From the image analysis, it shows that IDH1 mutation, EGFR expression, MGMT methylation, and p53 WT are beneficial for prognosis and are statistically significant. In contrast, ATRX positivity does not significantly impact patient prognosis.

Linear regression revealed no significant correlation between age and survival; however, tumor volume was inversely correlated with survival time. Figure 5 demonstrates the relationship between genetic markers and survival via KM curves. IDH1 mutation, EGFR+, MGMT methylation, and wild-type p53 (p53 WT) were significantly associated with favorable prognosis. ATRX + had no significant effect on survival.

**Figure 6:**
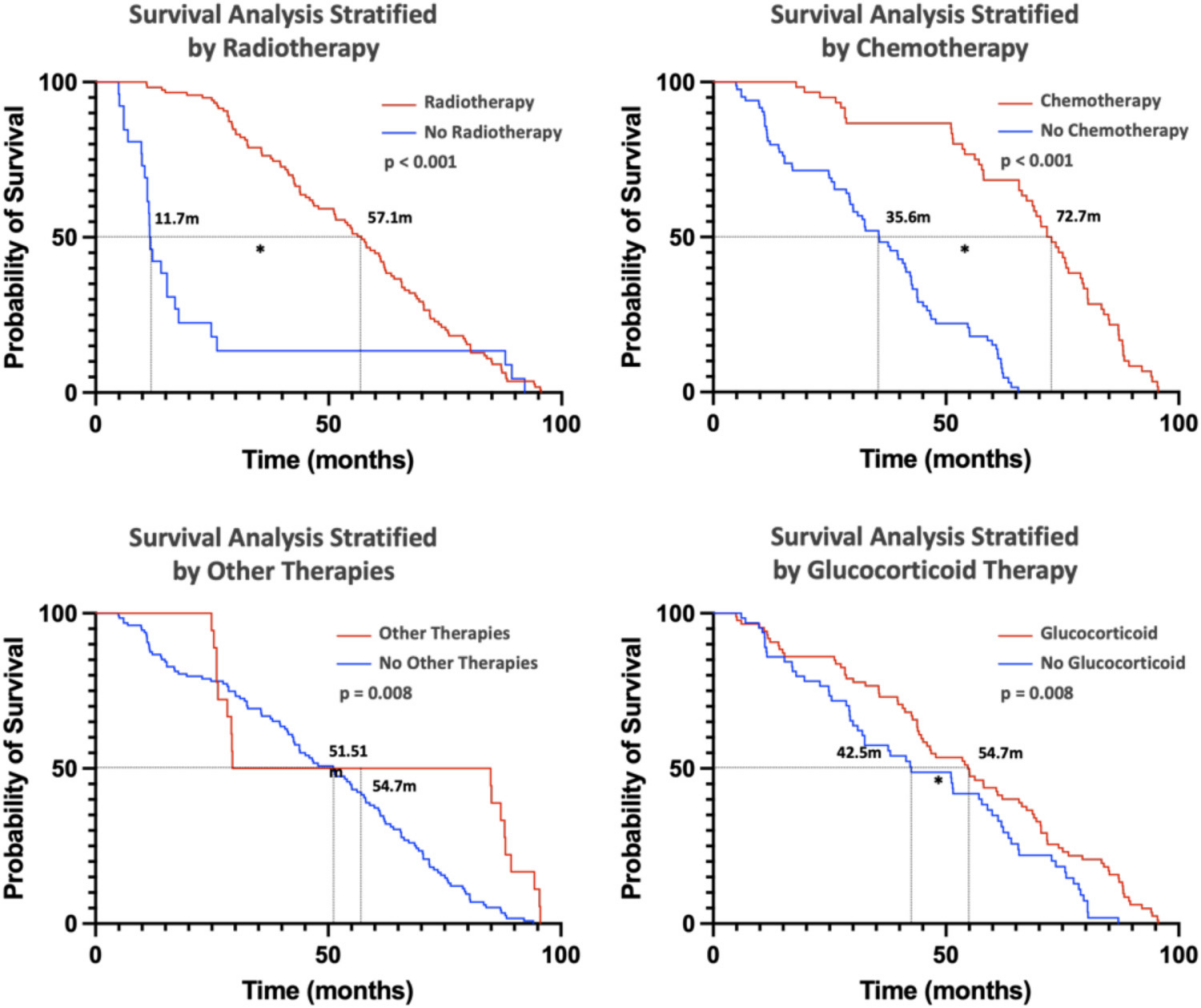
This figure primarily illustrates the impact of different treatments on patient prognosis. (Kaplan–Meier curves were used for the analysis, with * indicating significance at *p* < 0.05). Radiotherapy, chemotherapy, other therapies, and glucocorticoid therapy are all beneficial for patient prognosis.

Additionally, the impact of treatment modalities on survival was assessed (Figure 6). Radiotherapy, chemotherapy, other treatments (including traditional Chinese medicine and targeted therapies), and corticosteroid use were all associated with improved prognosis. These findings highlight the critical role of clinical features, genetic markers, and treatment strategies in determining survival outcomes for pHGG patients.

## Discussion

This study aims to systematically analyze the risk factors for VTE in pHGG patients through a multi-center retrospective analysis, and to explore factors related to prognosis. The study ultimately enrolled 168 patients who met the inclusion criteria, with an average age of 9.87 ± 3.67 years, and 37.5% of patients experienced VTE. Anticoagulation therapy, IDH1 mutation, MGMT methylation, radiotherapy, chemotherapy, and prolonged bed rest can reduce the risk of VTE, while larger tumor volume, grade 4 glioma, EGFR positivity, p53 mutation, glucocorticoid therapy and CVCP increase the risk of VTE. The median survival time for all patients was 51.4 months, and the occurrence of VTE had a negative impact on patient prognosis.

While any malignancy can predispose to VTE, the highest risks are observed in gliomas, and HGGs have unique VTE risk profiles (15, 16). Treatment often involves multiple chemotherapeutic agents due to the vascular nature of gliomas, which may further disrupt coagulation homeostasis and complicate anticoagulation management (17). There are differences in the occurrence of VTE between pediatric and adult patients with HGG: Firstly, the coagulation physiology in children significantly differs from that in adults (18). For instance, children tend to have lower levels of antithrombin III (AT3) and protein C, which may impact the efficacy and safety of anticoagulant therapy. Additionally, their faster metabolism and higher drug clearance rates necessitate careful dosage adjustments and monitoring. Furthermore, pHGG patients often exhibit neurological dysfunction and become bedridden at an earlier stage; therefore, the prevention and management of VTE risk factors in pHGG patients present unique challenges (19, 20).

Previous studies have discussed the occurrence of VTE in pHGG relatively less frequently. This multi-center retrospective study aims to assess the clinical characteristics, VTE risk factors, and survival outcomes of pHGG patients to identify risk factors and guide anticoagulation strategies. Overall, the identified risk factors are similar to those in the adult HGG population. Patient-specific factors (such as age and gender) are not significantly associated with VTE risk, but anticoagulant therapy is a significant protective factor supporting its clinical utility. Among tumor genetic mutations, IDH1 mutations and MGMT methylation are negatively correlated with the occurrence of VTE, and these two genetic mutations have also been proven to be negatively correlated with the occurrence of VTE in adult gliomas. In patients with IDH1-mutated gliomas, the expression levels of tissue factor (TF) and podoplanin (PDPN) are lower, and high expression of these proteins is associated with increased VTE risk (21). Furthermore, IDH1 mutations may inhibit TF expression by increasing DNA and histone methylation, thereby reducing VTE risk (22). Similarly, these two genetic mutations have been shown to improve patient prognosis. However, there are relatively fewer studies related to MGMT methylation. Additionally, common EGFR amplification and p53 mutations in gliomas lead to an increased incidence of VTE in children, with the former possibly related to the regulation of increased TF expression (21), while the latter may indirectly affect VTE risk by affecting cell cycle regulation and the tumor microenvironment. For example, p53 mutations may be related to tumor invasiveness and inflammatory responses, both of which may increase VTE risk (21).

Standard treatments (such as radiotherapy and chemotherapy) play an important role in improving the prognosis of glioma patients, and have significant improvement effects on patients' neurological dysfunction and limb activity, and can reduce the risk of VTE (23). During radiotherapy, by avoiding key brain areas such as the hippocampus, it is possible to protect patients' cognitive function without reducing prognosis (24). In addition, radiotherapy and chemotherapy can not only act directly on tumor cells but also indirectly improve patients' neurological function by reducing tumor burden, thereby improving patients' quality of life (25). In terms of limb activity, standard treatment helps patients recover limb strength and coordination by controlling tumor growth and reducing brain edema. In addition, during standard treatment, patients usually receive rehabilitation training, which further promotes the recovery of limb function and reduces the risk of VTE (26). Other anti-tumor treatments included in this study, including traditional Chinese medicine treatment and targeted treatment, have no significant effect on the occurrence of VTE.

Furthermore, glucocorticoid treatment is also associated with an increased risk of VTE, which is consistent with previous studies (27). Studies have shown that during the use of glucocorticoids, the risk of VTE in patients is significantly increased, and this risk is highest in the first few weeks after the start of treatment. For example, a study found that the incidence of the first VTE during glucocorticoid treatment was 3.51 times higher than during non-use periods (28). The use of glucocorticoids may directly increase the risk of VTE through multiple mechanisms, including promoting coagulation and inhibiting fibrinolysis (29). Therefore, when using glucocorticoid treatment, the impact on VTE risk should be fully considered, and appropriate preventive measures should be taken. However, for glioma patients, the use of glucocorticoids to control cerebral edema and neurological symptoms is inevitable, and whether the replacement of existing drugs (such as bevacizumab) can further improve the occurrence of VTE requires further research.

In this study, we unexpectedly found that prolonged bed rest was associated with a reduced risk of VTE, which contradicts the common belief that immobilization increases the risk of VTE (30). Possible explanations include: patients who are bedridden for extended periods may receive more aggressive prophylactic anticoagulation therapy and closer medical monitoring, thereby reducing the risk of VTE; moreover, once their condition stabilizes, these patients may be encouraged to engage in early mobilization to promote blood circulation and reduce thrombus formation (31, 32). However, these explanations require further research for validation. It is important to note that our study results may be influenced by the selection of sample size, especially if the sample size is small or there is selection bias, the observed associations may not be generalizable.

This study has several limitations. The retrospective design may introduce selection bias, and the small sub-group sample size (especially for genetic analysis) may limit generalizability. Some subgroup analyses, such as different chemotherapy regimens and different genetic mutations, may bring certain biases due to the small sample size of subgroups. In addition, due to the availability of data, we were unable to analyze the correlation between comorbidities and VTE occurrence. Future larger-scale prospective studies and more refined treatment classification validation are needed.

## Conclusion

This study the highlights risk factors for VTE in pHGG patients, emphasizing the need for tailored prevention and treatment strategies. The findings underscore the importance of clinical characteristics, genetic profiles, and treatment modalities in managing VTE and improving survival outcomes. Future prospective studies with larger cohorts are recommended to validate these findings and refine therapeutic approaches.

## Data Availability

The original contributions presented in the study are included in the article/Supplementary Material, further inquiries can be directed to the corresponding author.
