# Supplementary material for: Venous thromboembolism risk factors in pediatric patients with high-grade glioma: a multicenter retrospective study
Source: Front Pediatr. 2025 Aug 12;13:1595223. doi: 10.3389/fped.2025.1595223 (PMC12378311; doi:10.3389/fped.2025.1595223)
Supplement: Supplementary file 1 [file Table1.docx]

| **Table S1: Distribution of Chemotherapy Regimens in Pediatric Glioma Patients with and without VTE** | | | |
| --- | --- | --- | --- |
| **Chemotherapy Regimen** | **VTE (n=18)** | **NO VTE(n=41)** | p |
| **Regimen A (Carboplatin + Vincristine)** | 2 (11.1%) | 3 (7.3%) | 0.68 |
| **Regimen B (Carboplatin + Vincristine + Temozolomide)** | 2 (11.1%) | 6 (14.3%) | 0.71 |
| **Regimen C (Carboplatin + Vincristine + Bevacizumab)** | 4 (22.2%) | 10 (24.4%) | 0.84 |
| **Regimen D (****Temozolomide Monotherapy)** | 7 (38.9%) | 12 (29.3%) | 0.44 |
| **Regimen E (Temozolomide + Bevacizumab)** | 3 (16.7%) | 7 (17.1%) | 0.94 |
| **Regimen F (Other Regimens)** | 0 (0%) | 3 (7.3%) | 0.12 |
